# Modification of Eutectic Si in Al-Si-(Ba) Alloy by Inducing a Novel 9R Structure in Twins

**DOI:** 10.3390/ma11071151

**Published:** 2018-07-06

**Authors:** Xuechao Sha, Xuefei Chen, Huiyan Ning, Lirong Xiao, Dongdi Yin, Lin Mao, Jiang Zheng, Hao Zhou

**Affiliations:** 1 Beijing Key Lab of Microstructure and Property of Advanced Materials, Beijing University of Technology, Beijing 100124, China; shaxuechao@emails.bjut.edu.cn (X.S.); xiaolr620@126.com (L.X.); hzhou511@njust.edu.cn (H.Z.); 2 Nano and Heterogeneous Materials Center, School of Materials Science and Engineering, Nanjing University of Science and Technology, Nanjing 210094, China; 3 State Key Laboratory of Nonlinear Mechanics, Institute of Mechanics, Chinese Academy of Sciences, 15 Beisihuan West Road, Beijing 100190, China; 4 School of Mechanical and Electrical Engineering, Heilongjiang Institute of Technology, Harbin 150050, China; ninghuiyan2000@163.com; 5 School of Materials Science and Engineering, Harbin University of Science and Technology, Harbin 150040, China; 6 Key Laboratory of Advanced Technologies of Materials, Ministry of Education, School of Materials Science and Engineering, Southwest Jiaotong University, Sichuan 610031, China; ahnydd@swjtu.edu.cn; 7 Shanghai Institute for Minimally Invasive Therapy, School of Medical Instrument and Food Engineering, University of Shanghai for Science and Technology, Shanghai 200093, China; linmao@usst.edu.cn; 8 International Joint Laboratory for Light Alloys (Ministry of Education), College of Materials Science and Engineering, Chongqing University, Chongqing 400044, China

**Keywords:** Ag-Si alloy, modification, twinning morphology, 9R structure, HRTEM

## Abstract

The change of twinning morphology plays an important role in the modification of Al-Si alloys, which are widely used in industrial applications. However, the interpretation of this change is still insufficient. In this work, the microstructure of twins was investigated in two kinds of Al-Si alloys with different additions of Ba using high-resolution transmission electron microscopy (HRTEM). Unlike the normal {111} twin that exists in Ba-free alloy, discontinuous twins and multiple twins were observed in the Ba-containing alloy. In addition, the 9R structure formed by the dissociation of twins was firstly observed at the turning of discontinuous twins and the intersection of multiple twins in Al-Si alloys.

## 1. Introduction

Al-Si alloys have been widely applied in automotive and aircraft application, due to the good castability, favorable mechanical properties, low weight, excellent abrasion resistance, good corrosion resistance, and low cost [1,2,3,4,5,6]. The formation mechanism of coarse eutectic Si has been reported as twin plane re-entrant edge (TPRE) [7,8,9], which proposes that Si grain tends to grow fast at the re-entrant edge along a single <112> direction. It has been confirmed that the refinement and passivation of eutectic Si is an effective way to improve the mechanical properties of Al-Si alloys [10,11,12]. The normal methods to modify eutectic Si are as follows: adding modifying elements [10], rapid solidification [13,14], mechanical or electromagnetic stirring [15,16], ultrasonic treatment [17,18], etc. Adding a modifying element (Na, Sr, etc.) is considered to be the most effective method in industrial production.

A variety of modifying elements, including Na, Sr, Ba, Ca, and Eu, have been widely studied, which show a significant modifying effect on the morphology of eutectic Si. The refinement of eutectic Si from coarse plate-like into fine fibrous leads to a significant improvement on both the strength and ductility of the alloys. The mechanism named impurity-induced twinning (IIT) is generally accepted to explain the modification induced by modifying elements [9,19,20]. The IIT mechanism suggests that the modifying elements are more easily adsorbed at the {111} growth step of eutectic Si during solidification. The different sizes of the adsorbed modifying atom and Si atom will change the local stacking sequence, promoting the formation of multiple twins. These twins can limit the fast growth of the Si grain and provide more growth directions, resulting in the modification of eutectic Si. According to the IIT mechanism, a different modification effect relates to the different atomic radius ratio of modifying an atom and a Si atom. The ideal ratio to induce the formation of twins is considered *r*/*r*_Si_ = 1.646. Actually, this ideal ratio cannot interpret all of the observations accompanying modification. For example, the addition of Yb to hypoeutectic Al-Si alloys only refines, rather than modifies, the eutectic Si, even though Yb possesses a favorable atomic radius ratio (*r*_Yb_/*r*_Si_ = 1.646) [21,22]. Recent studies have shown that the investigation of the twinning microstructure in modified eutectic Si is necessary for understanding the modification mechanism. Eu, Na, and Sr-rich clusters have been observed at the intersection of multiple {111} twins in corresponding modified Al-Si alloys [22,23]. So far, although Ba has a good modification effect with a larger ratio at 1.86 [20,24], the microstructural characteristics of twinning in casting Al-Si alloys modified with Ba has never been clearly studied.

In this work, two kinds of Al-Si alloys with different additions of Ba were comparatively studied to investigate the change of twinning morphology. The microstructure of the twins in these alloys were observed using high-resolution transmission electron microscopy (HRTEM). The discontinuous twins and multiple twins were found in the Ba-containing alloy. In addition, the structure named 9R (the stacking order of the crystal is nine-layer repeat sequence) formed by the dissociation of twins was firstly observed at the turning of discontinuous twins and the intersection of multiple twins in casting Al-Si alloys.

## 2. Material and Methods

Chemical compositions of the two alloys are Al-7Si (wt. %), except for 50 ppm Ba. The alloys were prepared from high purity Al, Ba, and Al-18Si master alloy in a 5-kW crucible electric resistance furnace. Fluxes were added to refine the melt at 720 °C; then, the melting temperature was held at 735 ± 5 °C, and the molten metal was degassed using high purity argon degas. The chemical compositions were determined by an inductively coupled plasma atomic emission spectrum (ICP-AES) apparatus.

HRTEM samples were cut from the center of the as-cast ingots, and then polished and dimpled until the thickness reached ~30 µm. Ion milling was carried out using a Precision Ion Polishing System (PIPS model 695, Gatan, Beijing, China) at −25 °C to avoid the damage to the microstructure. The HRTEM study was performed in a Titan^TM^ G2 60-300 TEM (FEI, Beijing, China) with Probe-Spherical aberration performed at 300 kV.

## 3. Results and Discussion

**Figure 1:**
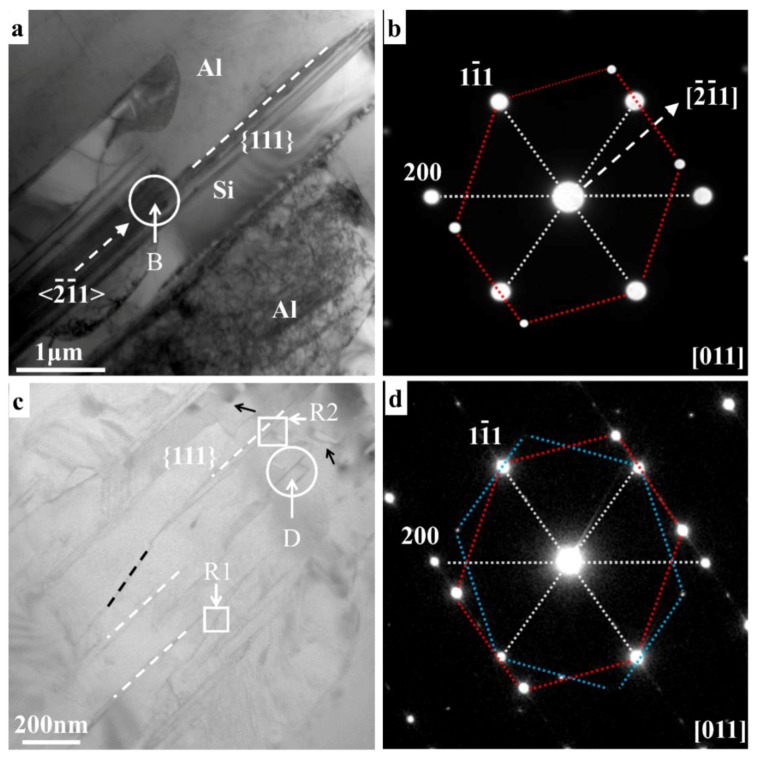
Low magnification TEM images and corresponding selected area diffraction pattern (SADP) of eutectic Si in different Al-Si alloys, viewed from the [011] zone axis, (**a**) the eutectic Si in Al-7Si alloy; (**b**) the SADP taken from the white circle B; (**c**) the eutectic Si in Al-7Si-50 ppm Ba alloy; (**d**) the SADP taken from the white circle D.

Figure 1 shows the low magnification TEM images of the two alloys, viewed from the [011] zone axis. A series of typical parallel strip-shape twinning are observed in eutectic Si grain in Ba-free alloy, as shown in Figure 1a. Selected area diffraction pattern (SADP) taken from the white circle B (in Figure 1a) indicates that these twins have a {111} twin relation and were grown along the <$\bar{2}\bar{1}$1> direction, which is also the long axis of eutectic Si grain, as shown in Figure 1b. It suggests that the growth of eutectic Si in unmodified Al-Si alloy is based on the TPRE mechanism. In contrast, three types of twinning are observed in eutectic Si grain in Ba-containing alloy, as shown in Figure 1c. The SADP (Figure 1d) taken from the white circle D (in Figure 1c) shows that they are normal {111} twins, discontinuous {111} twins, and multiple {111} twins, as marked by white dash lines, black dash lines, and black arrows, respectively. The above observations indicate that the addition of Ba can change the twinning morphology in eutectic Si.

**Figure 2:**
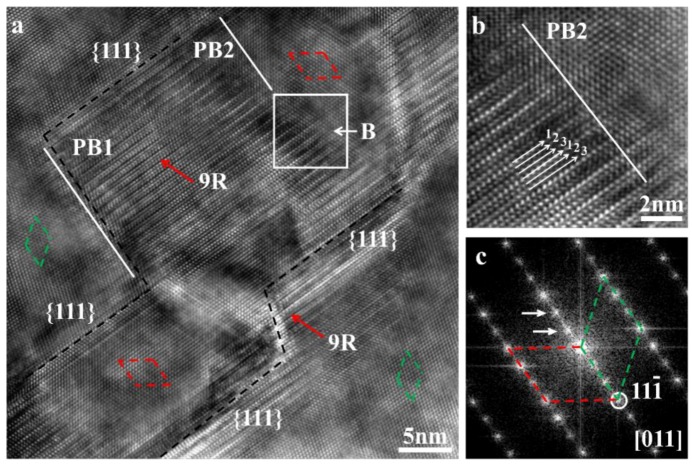
The 9R is located at the turning of discontinuous twin boundaries. (**a**) High-resolution transmission electron microscopy (HRTEM) image of region R1 in Figure 1c; (**b**) The magnification image of the white square B; (**c**) The corresponding Fourier transformation (FFT) pattern of Figure 2a.

To find the modification mechanism of twinning morphology induced by the addition of Ba, it is necessary to investigate the microstructure of discontinuous and multiple twins. The HRTEM image of the region R1 (Figure 1a) with discontinuous twins is shown in Figure 2a, as viewed from the [011] zone axis. Two discontinuous twin boundaries marked by black dash lines can be observed, and the distance between them is alterable. Figure 2c shows the corresponding Fourier transformation (FFT) pattern. Two group diffraction spots that are symmetrical to the (11$\bar{1}$) spot have been marked by green and red dash lines respectively, indicating the {111} twin relation. It is noted that a special structure is located at the turning of the discontinuous twin boundaries, marked by red arrows. This structure is consistent with the 9R, which has been observed in deformed FCC bulk metal, such as Cu, Ag etc. [25,26,27]. However, it has never been reported in eutectic Si modified by modifying elements. The features of 9R are as follows. (1) The 9R is composed of a repeated unit with the periodicity of three adjacent {111} planes, as shown in Figure 2b. (2) The 9R is separated from the matrix by two phase boundaries, which are marked by PB1 and PB2, and the {111} twin boundaries, as shown in Figure 2a. (3) The 9R induces the extra diffraction spots at the positions of 1/3{$\bar{1}\bar{1}$1} and 2/3{$\bar{1}\bar{1}$1}, which are marked by white arrows in Figure 2c.

**Figure 3:**
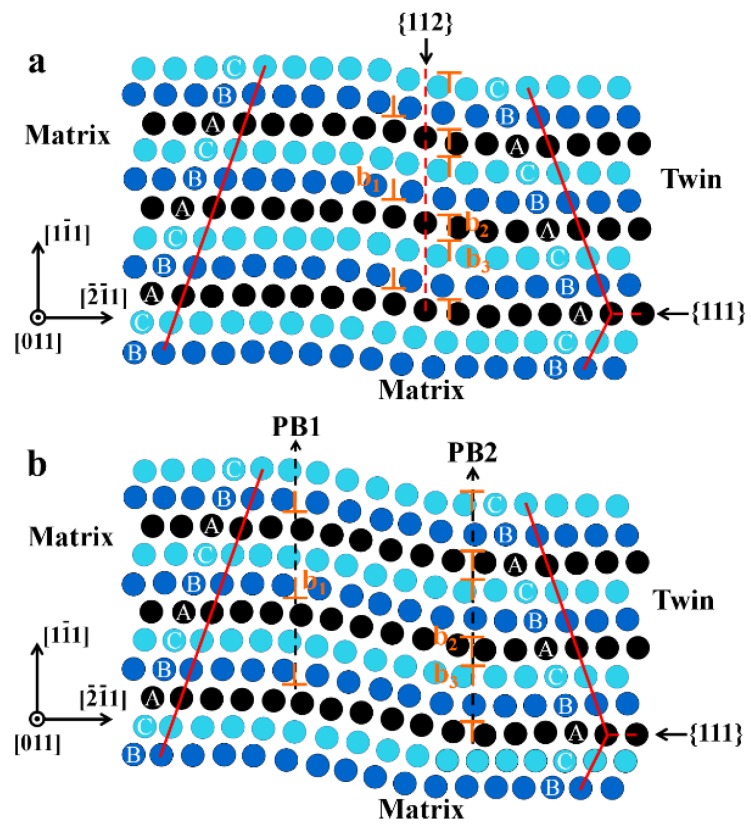
The schematic of the formation process of 9R. (**a**) The structure of the twin that is formed by the cooperative glide of three repeated partial dislocations; (**b**) The structure of 9R that is formed by the dissociation of twins.

The 9R is known to be formed by the dissociation of twinning in FCC metals [26,27,28]. Figure 3 shows the schematic of the formation process of 9R. The {111} twins in FFC metals are known to be formed by the glide of Shockley partial dislocations on successive slip planes [29,30]. One type of twin is formed by the cooperative glide of three partials (CSTP), which was proposed by Wang et al. [26,28,31] and Li et al. [32]. This twin consists of a series of partial dislocations with a repeatable sequence b_2_-b_1_-b_3_ on every (111) plane, as shown in Figure 3a. The b_1_ is a pure-edge partial dislocation. The b_2_ and b_3_ are mixed partial dislocations with opposite sign of screw components. The opposing signs of the screw components make b_2_ and b_3_ difficult to move. In contrast, b_1_ can deviate from the original position by several atoms in order to achieve equilibrium status under a driving force. It is noted that the driving force in deformed materials is mainly applied stress, but in as-cast materials, it is induced by the temperature gradient during solidification [33]. When the equilibrium distance is greater than the scale of one dislocation core, it can be considered that the twin has dissociated into 9R with two PBs(phase boundary), as shown in Figure 3b. Owing to the immobile PB2 formed by b_2_ and b_3_, the extension of twins that have dissociated into 9R is limited. However, the remaining twins still can extend along the original direction, causing the formation of discontinuous twins.

**Figure 4:**
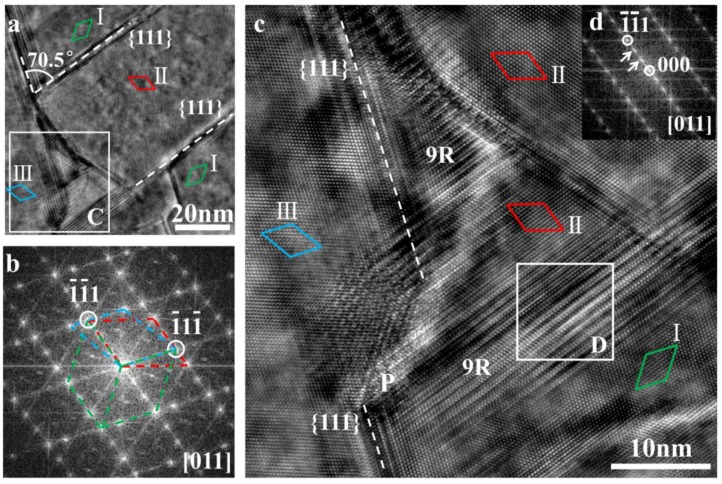
The 9R structure is located at the intersection of multiple {111} twins in modified eutectic Si. (**a**) The HRTEM image of the region R2 in Figure 1c with multiple twins; (**b**) The corresponding FFT pattern of (**c**); (**c**) The magnification image of region C; (**d**) The FFT pattern of region D.

The 9R is also observed at the intersection of multiple twins in modified eutectic Si, as shown in Figure 4. Figure 4a is the TEM image of multiple twins taken from the region R2 (in Figure 1b) along the [011] zone axis. According to the corresponding FFT pattern (Figure 4b), three groups of diffraction spots can be found and marked by green, red, and blue dash lines, which are named as I, II, and III, respectively. The I and II are symmetrical of the {111} spot, which is the same as I and III, indicating the multiple {111} twin relations. Figure 4c is the HRTEM image of region C in Figure 4a. The local FFT pattern (Figure 4d) was taken from region D in Figure 4c, indicating the existence of 9R at the intersection of multiple twins. According to the different stacking sequence between 9R and the matrix mentioned in Figure 3, it can be considered that the 9R induces the formation of multiple twins, which is similar to the effect of modifying the atom in the IIT mechanism.

In addition, the 9R structure is frequently reported in FCC metals with low stacking fault energy (SFE) [34,35,36,37]. So, it can be concluded that the SFE of modified eutectic Si is decreased by Ba addition. The lower SFE means a smaller resistance for the glide of PB1; then, the 9R is easier to form. Lower SFE is also conducive to the formation of twins.

## 4. Conclusions

To correctly interpret the change of twin morphology in the modified Al-Si alloy, the microstructure of twins in Ba-containing alloy was observed using HRTEM. The results can be summarized as follows:

1. In the Ba-free Al-Si alloy, the size of the eutectic Si is large, which is a result of the uniaxial growth of strip-shaped twins in Si grains. Meanwhile, the morphology of twins exhibits a combination of normal twins, discontinuous twins, and multiple twins in Ba-containing Al-Si alloy. Ba addition modified the eutectic Si from coarse plate-like into fine fibrous, which is the optimized microstructure for both strength and ductility.
2. A novel 9R structure is observed at the turning of discontinuous twins, which is first reported in the cast Al-Si alloy. The phase boundary of the 9R structure is a large mismatched region that leads to the growth of twins with steps to form discontinuous twins.
3. The 9R structure is also observed at the intersection of multiple twins. It indicates that the formation of multiple twins may induced by a different stacking sequence between 9R and the matrix, which is similar to the IIT (impurity-induced twinning) mechanism.
